# Performance Evaluation of an Immersive Virtual Reality Application for Rehabilitation after Arthroscopic Rotator Cuff Repair

**DOI:** 10.3390/bioengineering10111305

**Published:** 2023-11-10

**Authors:** Arianna Carnevale, Ilaria Mannocchi, Emiliano Schena, Marco Carli, Mohamed Saifeddine Hadj Sassi, Martina Marino, Umile Giuseppe Longo

**Affiliations:** 1 Fondazione Policlinico Universitario Campus Bio-Medico, Via Álvaro del Portillo, 200, 00128 Roma, Italy; arianna.carnevale@policlinicocampus.it (A.C.); martinamarino.dr@gmail.com (M.M.); 2 Department of Industrial, Electronic and Mechanical Engineering, University of Roma Tre, Via Vito Volterra, 62, 00146 Roma, Italy; ilaria.mannocchi@uniroma3.it (I.M.); marco.carli@uniroma3.it (M.C.); mohamedsaifeddine.hadjsassi@uniroma3.it (M.S.H.S.); 3 Unit of Measurement and Biomedical Instrumentation, Department of Engineering, Università Campus Bio-Medico di Roma, Via Álvaro del Portillo, 21, 00128 Roma, Italy; e.schena@unicampus.it; 4 Research Unit of Orthopaedic and Trauma Surgery, Department of Medicine and Surgery, Università Campus Bio-Medico di Roma, Via Álvaro del Portillo, 21, 00128 Roma, Italy

**Keywords:** shoulder, rotator cuff, virtual reality, rehabilitation, orthopaedics, wearable systems

## Abstract

Few studies have evaluated the effectiveness of shoulder rehabilitation in virtual environments. The objective of this study was to investigate the performance of a custom virtual reality application (VR app) with a stereophotogrammetric system considered the gold standard. A custom VR app was designed considering the recommended rehabilitation exercises following arthroscopic rotator cuff repair. Following the setting of the play space, the user’s arm length, and height, five healthy volunteers performed four levels of rehabilitative exercises. Results for the first and second rounds of flexion and abduction displayed low total mean absolute error values and low numbers of unmet conditions. In internal and external rotation, the number of times conditions were not met was slightly higher; this was attributed to a lack of isolated shoulder movement. Data is promising, and volunteers were able to reach goal conditions more often than not. Despite positive results, more literature comparing VR applications with gold-standard clinical parameters is necessary. Nevertheless, results contribute to a body of literature that continues to encourage the application of VR to shoulder rehabilitation programs.

## 1. Introduction

The application of Virtual Reality (VR) as a diagnostic and therapeutic tool is emerging as a viable alternative for musculoskeletal rehabilitation of the upper limb [1,2,3]. Unlike in orthopedic rehabilitation, VR has been widely used in the field of neurorehabilitation, such as stroke, brain injury, and cerebral palsy [4,5,6,7,8,9]. On the other hand, several studies have been conducted on the effectiveness and application of VR in orthopedics for educational and training purposes in shoulder, elbow, knee, hip, or ankle surgery [1,10,11,12,13,14].

VR technology is an advanced human–computer interface simulating a virtual environment where people can move and interact with unreal objects [15,16]. Based on the degree of immersion in the virtual environment, VR systems can be classified into non-immersive, semi-immersive, or immersive [16,17]. The most recent immersive VR devices offer a three-dimensional stereoscopic vision through a head-mounted display (HMD) and input devices (VR controllers), in which users are immersed and can interact with the virtual environment [18,19]. Among VR devices, Oculus Quest 2 showed good performance and accuracy during controlled translational and rotational displacements, establishing itself as an excellent VR device candidate for use during shoulder rehabilitation exercises [2]. Given that VR focuses users’ attention on the perceived experience, designing applications tailored to the final population and context of use is crucial. VR applications could be extremely interesting and impactful in shoulder rehabilitation. Indeed, in an increasingly digitized society, VR can be considered an adjunct to standard physiotherapy to increase patients’ motivation, compliance, and engagement during therapy sessions. However, delineating a perfect balance between the efficiency of rehabilitation exercises in the virtual environment intended for patients with rotator cuff (RC) diseases, ease of use, and engagement is challenging.

RC diseases have a high incidence in the working population, entailing pain, reduced range of motion (ROM), and absence from work [20,21,22]. Treatments of RC tears can be conservative or surgical [23,24,25]. The surgical approach, eventually selected in patients unresponsive to conservative treatment, requires a postoperative phase in which the perfect balance between immobilization and mobilization is crucial to preserve the integrity of the repaired tendon and, at the same time, avoid shoulder stiffness [21]. Shoulder rehabilitation after rotator cuff repair (RCR) aims to restore full painless shoulder ROM, gradually introducing muscle-strengthening exercises under the supervision of expert physiotherapists who monitor and tune exercises’ difficulty based on the patient’s recovery [26].

In the orthopedic rehabilitation field, few studies have evaluated the effectiveness of kinematic intervention in virtual environments in patients with shoulder stiffness, subacromial impingement syndrome, scapular dyskinesis, and RC diseases [3,27,28,29,30]. Chang et al., in a mono-centric randomized controlled trial with patients undergoing RCR, compared a control group following conventional home rehabilitation to the experimental one, which used an augmented reality (AR)-based digital healthcare system [28]. Overall, the authors support the effectiveness of the developed AR-based post-surgical rehabilitation following arthroscopic RCR [28]. In a single-blind randomized trial, the efficiency of video-based game rehabilitation exercises and closed kinetic chain was investigated related to conventional care in patients with partial RC tears, showing significant improvement in the outcomes of the experimental groups [31]. To the best of our knowledge, regarding VR applications designed for the rehabilitation following RCR, only one study presented an approach more similar to ours [32]. Baldominos et al. tested a custom application reproducing abduction and adduction movements with four professionals of physiotherapy after RCR, using the VR glasses Oculus Rift DK2 in association with the motion-tracking system Inter RealSense [32]. However, none of the previous studies mention evaluating the performance of developed VR applications against pre-established criteria inherent in purely clinical guidelines and staged ROM goals.

In this study, we propose an immersive VR application (VR app) designed for the rehabilitation of patients after RC arthroscopic repair, following recommendations developed by the American Society of Shoulder and Elbow Therapists [33]. The VR app includes four propaedeutic levels reproducing increasing levels of ROM similar to what is required during rehabilitation pathways. The objective of this study was to investigate the performance of the custom VR app by comparing the movements performed during the different levels of the VR app with a stereophotogrammetric system considered the gold standard.

## 2. Materials and Methods

### 2.1. Population and Equipment

Five volunteers (male/females—2/3; mean age ± standard deviation—24.4 ± 3.0 years old; body mass—67.6 ± 16.9 kg; height—1.70 ± 0.10 m) were enrolled in this study. Volunteers were considered eligible for participation in the study if all of the following criteria were met: age ≥18 years old, no shoulder musculoskeletal disorders, and no previous shoulder surgery. All but one of the participants were right handed. The experimental sessions were executed in the Laboratory of Motion Analysis at Fondazione Policlinico Universitario Campus Bio-Medico in Rome. Before each experimental session, all volunteers were briefed on the study’s purpose and mode. The experiments began after a comprehensive understanding and delivery of the dated and signed consent. Ethical approval was granted by the local Ethical Committee (protocol code: 120/121 OSS ComEt UCBM).

The VR device used in this study was the Oculus Quest 2 (OQ2, Meta Platforms Technologies). Volunteers wore the HMD and, through two controllers housed at the ends of a double-handled stick (Beat Saber Handle), were able to interact and move within the virtual environment created ad hoc for the specific application. Without the use of external sensors, the VR system can track the movements of the head and hands and replicate them in the virtual world.

Shoulder kinematics was recorded by the Qualisys™ motion capture system (Qualisys AB, Gothenburg, Sweden). In each experimental session, photo-reflective markers (diameter, 8 mm) were placed on the following anatomical markers: incisura jugularis, processus xiphoideus, C7 vertebra, T8 vertebra, and bilaterally on the acromioclavicular joint, coracoid process, trigonum spinae scapulae, angulus inferior, angulus acromialis, medial and lateral epicondyles, and radial and ulnar styloid [34]. The glenohumeral rotation center was estimated using the linear regression method [35]. Moreover, five rectangular-shaped clusters with four markers each were placed on the thorax and bilaterally on the upper arms and forearms to track segments during dynamic trials [36]. Ten Miqus M3 cameras recorded markers’ trajectories at 100 Hz.

### 2.2. Game Design and Development

A custom VR application was developed in Unity3D (version 2020.17) using the Oculus Integration Package. The game was designed considering the recommended rehabilitation exercises following arthroscopic RCR [33]. A collection of C# scripts and free Unity components have been used and implemented to develop different functions in the VR app.

**Figure 1:**
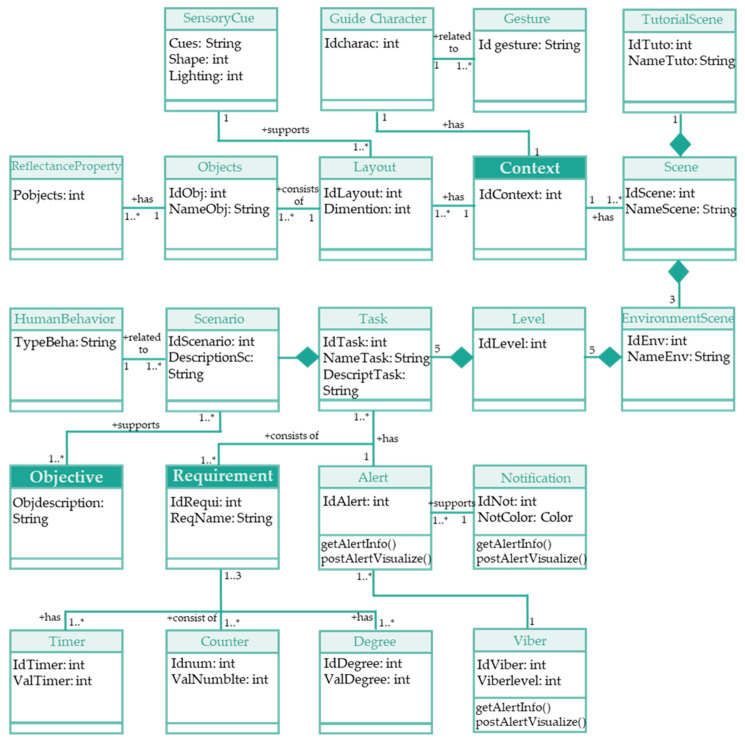
Meta model of the developed immersive virtual reality application. “*” means the variable number of elements.

The different steps taken to design the proposed rehabilitation in a VR scenario were specifications of requirements, context, and objectives. Figure 1 presents the meta model describing the specifications of the developed application, defining the collection of objects and their relationship. Each box in Figure 1 is an object with the properties contained in the same box. The boxes, which have a more intense color (Context, Objective, and Requirement), are the main objects around which the entire application was created. The lines connecting the various boxes show the relationship between the context, objective, and requirement elements. Finally, each layout consists of a collection of many objects indicated by a number next to the boxes.

#### 2.2.1. Specification of Requirements

**Figure 2:**
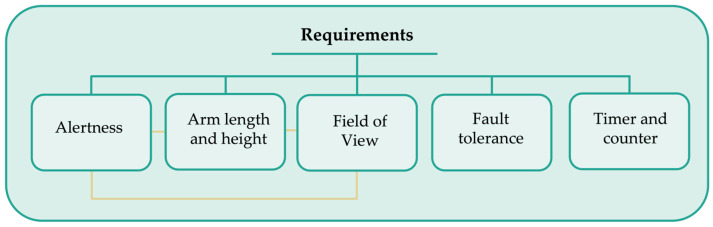
Specification of requirements of the developed immersive virtual reality application.

The following requirements were set in the developed VR app: alertness, arm length, height, vision degree, fault tolerance, timer, and counter (Figure 2). In this prototype of the VR app, the user can receive an alert signal about the position of the hand to notify the correct way to move the arm. Upper limb length and height can be set by pressing the trigger on the controller for ease of use by a heterogeneous population. Upper limb length and user height were key requirements for properly positioning all objects in the scene, thus improving the field of view (FOV). A controller vibration and an arrow of different colors (adapted to the degree of shoulder movement, user height, and arm length) can alert patients of right-hand movement and object position. The arrow is related to the angular degree and adapted to the arm length and user height. Fault tolerance is the ability to keep the service working without interruption by ensuring the reliability of different system components to assure an acceptable performance level tailored to specific application needs. Thus, an algorithm was developed to ensure that the controller of the VR device will work during the whole process, no matter if there is a lack of FOV and even in case of changing tasks or scenes while interacting with objects. A timer and counter were set for each scene to check the period of each training session and the number of performed movements.

#### 2.2.2. Specification of Context

To enhance the patient experience, proper scenes, layouts, and characters can be considered in a virtual environment. The layout could include sensory cues such as auditory feedback, the visual shape of the environment, and lighting that influence the user’s perception of the surroundings. The shapes and colors of the app, as well as the rate of lighting, are all variables that can strongly influence the user’s attitude and reaction. Specific cues were selected in the developed VR app to create the best possible user experience. Typical rehabilitation movements were replicated within a natural setting and with appropriate lighting conditions. These choices were guided by the need to improve task performance, enhance the performance area’s appearance, and positively affect patients’ perceptions and experience. In addition, the developed VR app shows a virtual physiotherapist at the beginning and end of each exercise to provide directions and motivational feedback to the patient.

#### 2.2.3. Specification of Objectives

An immersive and naturalistic scenario has been developed. In particular, the users can perform rehabilitation exercises in a natural world with objects like trees, apples, animals, and vegetables. Before task execution, patients may want to be trained on the use of the VR app. Thus, a VR starting tutorial was added to help users manage the device and understand the VR app’s functioning.

### 2.3. Movements Protocol

Following the setting of the play space and the user’s arm length and height, all participants performed four levels simulating some rehabilitative exercises after RCR. In the first phase of the experimental session, participants were asked to calibrate the system by measuring their arm length and height. These measures served as input for the developed scripts for positioning objects in the virtual environment attainable at each level with the predetermined ROM. During VR app development, developers of the Unity application used a common goniometer to fix the position of the virtual objects in the virtual environment. Table 1 reports the shoulder movements, ROM, and number of repetitions for each level.

The first level (Table 1) includes two sets of ten repetitions each of flexion movements in the range of 60°–90°, one set of ten repetitions of abduction movements up to 45°, and one set of eight repetitions of extra-rotations up to 20°. The second level (Table 1) includes two sets of ten repetitions each of flexion movements in the range 90°–120°, one set of ten repetitions of abduction movements in the range 45°–80°, one set of eight repetitions of extra-rotations in the range 20°–30°, and one set of eight repetitions of intra-rotations up to 20°.

The third level (Table 1) includes two sets of ten repetitions each of flexion movements in the range of 130°–155°, one set of ten repetitions of abduction movements in the range of 80°–120°, one set of eight repetitions of extra-rotations in the range 30°–45°, and one set of eight repetitions of intra-rotation in the range of 20°–50°. The fourth level (Table 1) includes two sets of twelve repetitions each of flexion movements in the range of 140°–ROM_MAX_, one set of twelve repetitions of abduction movements in the range of 120°–ROM_MAX_, one set of twelve eight repetitions of extra-rotations in the range of 45°–ROM_MAX_, and one set of twelve repetitions of intra-rotation in the range of 50°–ROM_MAX_.

**Figure 3:**
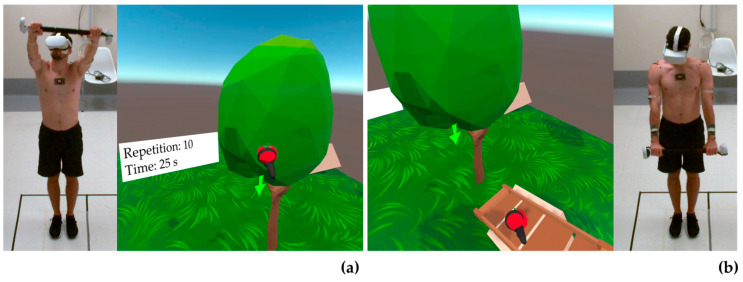
Representation of a healthy volunteer wearing the Oculus Quest 2, the Beat Saber Handle, and photo-reflective markers during flexion movement to reach the virtual target (**a**) and in the starting position (**b**). Arrows indicate the direction of movement.

**Figure 4:**
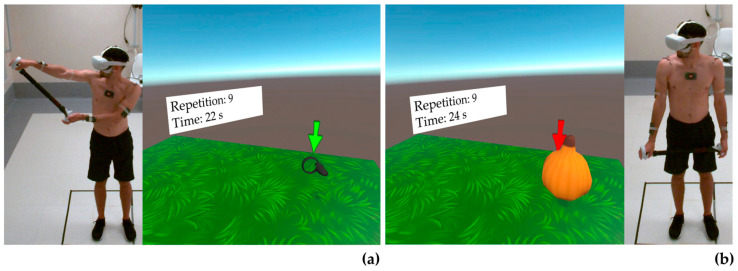
Representation of a healthy volunteer wearing the Oculus Quest 2, the Beat Saber Handle, and photo-reflective markers during abduction movement to reach the virtual target (**a**) and in the starting position (**b**). Arrows indicate the direction of movement.

**Figure 5:**
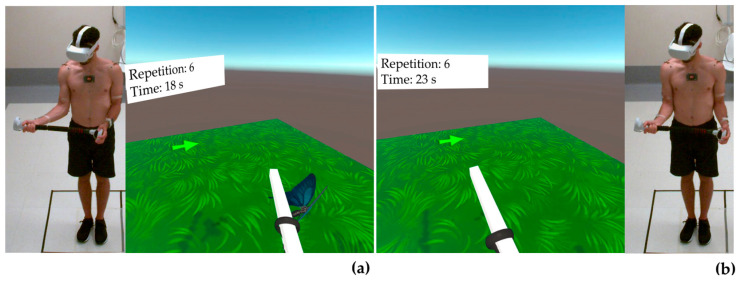
Representation of a healthy volunteer wearing the Oculus Quest 2, the Beat Saber Handle, and photo-reflective markers during external rotation to reach the virtual target (**a**) and in the starting position (**b**). Arrows indicate the direction of movement.

Figure 3, Figure 4 and Figure 5 show a healthy volunteer wearing the OQ2, the Beat Saber Handles, and photo-reflective markers while performing flexion, abduction, and external rotation movements, respectively. Internal rotation was not represented as it was identical to external rotation, except for the opposite direction of rotation.

### 2.4. Data Collection and Analysis

**Figure 6:**
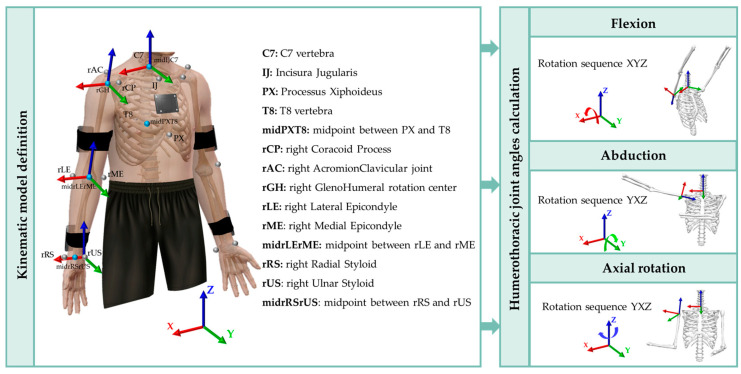
Schematization of shoulder joint angles calculation starting from the kinematic model definition (all markers are shown except trigonum spinae scapulae, angulus inferior, angulus acromialis). The local coordinate systems of the thorax, humeri, and forearms were based on the ISB recommendations [34]. Movements of the humerus relative to the thorax (humerothoracic joint angles) were quantified in terms of rotation sequences: XYZ for flexion (rotation around X) and YXZ for abduction (rotation around Y) and axial rotation (rotation around Z) [37,38].

All participants were asked to perform a static N-pose for 3 s for the kinematic model definition from the trajectories of the anatomical photo-reflective markers. Marker trajectories were collected and pre-processed (e.g., gap filling, labeling) with Qualisys Track Manager (QTM) software (v. 2023.1, build 7985). Then, markers’ trajectories were imported into Visual 3D (C-Motion, Inc., Germantown, WD, USA) for kinematic analysis through a custom pipeline. In Visual 3D, trajectories of anatomical markers during the static N-pose were used to define the local coordinate system and orientation of the thorax, humeri, and forearms [34]. Only data from the right side were further processed, as the VR app was designed only for the right side to date. The humerus 3D orientation was expressed relative to the thorax. In particular, the rotation sequences used to evaluate humerothoracic angles were abduction–adduction, flexion–extension, and internal–external rotation (YXZ sequence in Visual 3D) for movements in the frontal and transverse planes, and flexion–extension, abduction–adduction, and internal–external rotation (XYZ sequence in Visual 3D) for movements in the sagittal plane [37,38] (Figure 6).

The kinematic results obtained in Visual3D were exported in .mat format for subsequent analysis in MATLAB^®^ (version R2022b). Specifically, the waveforms of the flexion, abduction, internal, and external rotation angles were processed to identify the the peak values reached at each repetition. For each identified repetition, it was checked whether the corresponding peak value met the ROM condition set in the VR app, as follows:

$$InfCond=Peaks_{i}<Inferior\;Limit?\;\left\{\begin{array}{c} if\;InfCond==1,\;err_{i,inf}=\left|x_{GS,i}-x_{hyp,inf}\right|\\ if\;InfCond==0,\;err_{i,inf}=[\;] \end{array}\right.$$

$$SupCond=Peaks_{i}>Superior\;Limit?\;\left\{\begin{array}{c} if\;SupCond==1,\;err_{i,sup}=\left|x_{GS,i}-x_{hyp,sup}\right|\\ if\;SupCond==0,\;err_{i,sup}=[\;] \end{array}\right.$$

where InfCond and SupCond correspond to the inferior and superior bounds, respectively; $err_{i,inf}$ and $err_{i,sup}$ correspond to the absolute error at the *i*-th repetition for the lower and upper bounds of the established ROM, respectively; $x_{GS,i}$ is the angular value measured by the gold standard at the *i*-th repetition; $x_{hyp,inf}$ and $x_{hyp,sup}$ correspond to the lower and upper hypothesized values of the ROM, respectively. For each subject, the Mean Absolute Error for the inferior ($MAE_{inf}$) and superior ($MAE_{sup}$) conditions were computed as follows:

$$MAE_{inf}=mean(err_{inf})$$

$$MAE_{sup}=mean(err_{sup})$$

Let N,inf and N,sup be the total number of times the inferior and superior conditions were not met for all participants, respectively; then, the total Mean Absolute Error for the inferior ($MAE_{tot,inf}$) and superior ($MAE_{tot,sup}$) conditions were computed as follows:

$$MAE_{tot,inf}=mean(err_{N,inf})$$

$$MAE_{tot,sup}=mean(err_{N,sup})$$

where $err_{N,inf}$ and $err_{N,sup}$ are vectors 1 × N,inf and 1 × N,sup reporting the concatenated vector of errors made by all volunteers.

## 3. Results

Results for the first and second rounds of flexion, seen in Table 2 and Table 3, respectively, and for abduction (Table 4), displayed low values for mean absolute error (MAE) and for the number of times conditions were not met overall. Results for external rotation (Table 5) and internal rotation (Table 6) displayed total MAE values similar to those seen for abduction and flexion; however, the number of times conditions went unmet was significantly higher. This discrepancy in results is attributed to the inability of volunteers to perform isolated shoulder rotations and thus use trunk movements as compensation.

### 3.1. Flexion

#### 3.1.1. First Round of Flexion

Results for the first series of flexion are reported in Table 2. In level 1, volunteers were found to have a total inferior MAE equivalent to 6.4; this value gradually decreased with each level. Participants did not meet inferior conditions 8 times. Total superior MAE was found to be 2.9, and superior conditions were not met 5 times.

In level 2, volunteers had a total inferior MAE equivalent to 5.1 and a total of 15 unmet inferior conditions, representing the highest value of unmet conditions for both the first and second rounds of flexion. No error or unmet conditions were recorded for the superior limit.

Volunteers for level 3 had a total inferior MAE equivalent to 1.3 and a total of 4 unmet conditions. The total superior MAE was found to be 8.6, and conditions were unmet 3 times.

In level 4, volunteers only had one recorded instance of an unmet condition for inferior limit and a total inferior MAE equivalent to 0.8, which is the lowest error value for inferior flexion in both the first and second rounds.

#### 3.1.2. Second Round of Flexion

Results for the second series of flexion are reported in Table 3. In level 1, volunteers were found to have a total inferior MAE equivalent to 4.6, and participants did not meet inferior conditions 6 times. The total superior MAE was found to be 10.5, the highest for both rounds of flexion. The superior conditions were not met 6 times.

In level 2, volunteers had a total inferior MAE equivalent to 12.3, the highest value of mean error for inferior flexion overall. Participants did not meet inferior conditions 8 times. The total superior MAE was found to be 9.2, and only one volunteer (V4) contributed to this error value. In total, conditions were unmet 7 times.

In level 3, total inferior and superior MAE values were 4.8 and 2.1, respectively, while in both cases, conditions were not met twice.

In level 4, volunteers did not meet conditions 8 times and had a total inferior MAE equivalent to 2.6.

### 3.2. Abduction

Results for abduction are reported in Table 4. In level 1, abduction had the highest total superior MAE value and the highest number of times superior conditions were not met; values are 6.1 and 14, respectively.

In level 2, abduction had no error for the inferior condition, and volunteers never failed to meet conditions. For superior condition, the total superior MAE was found to be 4.7, and conditions were not met 4 times.

In level 3, abduction had a total inferior MAE of 8.6, the highest out of all levels, and conditions were not met 15 times. No mean error value was found, and no conditions were unmet for superior condition.

In level 4, abduction had a total inferior MAE of 6.6, and conditions were not met 22 times, which represents the highest value measured for both superior and inferior conditions during abduction.

### 3.3. External Rotation

Results for external rotation are reported in Table 5. In level 1, external rotation has a total superior MAE value of 6.5, while conditions were not met 32 times.

In level 2, external rotation has a total inferior MAE equivalent to 2.3, and volunteers did not meet conditions only once. The total superior MAE was found to be 2.6, and conditions were not met 12 times.

In level 3, external rotation has a total inferior MAE equivalent to 6.3, and volunteers did not meet conditions 16 times. The total superior MAE was found to be 2.6, and conditions were not met 5 times.

In level 4, external rotation had a total inferior MAE of 19.6, and conditions were not met 26 times; 19.6 represents the highest total MAE value for all recorded movements.

### 3.4. Internal Rotation

Results for internal rotation are reported in Table 6. In level 2, internal rotation has a total superior MAE value of 9.8, while conditions were not met 33 times. This was the highest number of times conditions were not met for all recorded movements.

In level 3, internal rotation has a total inferior MAE equivalent to 2.0, and volunteers did not meet conditions 6 times. For the superior condition, the internal rotation had no error and no conditions went unmet.

In level 4, internal rotation has a total inferior MAE equivalent to 8.0, and volunteers did not meet conditions 30 times.

## 4. Discussion

Among VR devices, OQ2 is an interesting tool for creating virtual scenarios in which patients can perform rehabilitative exercises according to clinical needs. The device allows the creation of specific games with different objectives in which patients can have an immersive and interactive experience. With OQ2 Touch controllers, patients’ hands and gestures can be directly conveyed into the game so that movements can appear in the VR intuitively and engagingly. The application of games and varied virtual scenarios has the potential to increase patient compliance levels, directly increasing the efficacy of rehabilitation programs [2,28,39]. Furthermore, machine learning algorithms and the modifiable nature of this tool combined can allow professionals to adapt rehabilitation programs to individual patients [39]. This application has the potential to increase the efficacy of rehabilitation for each individual, and it enables patients to become more engaged with the assigned tasks, given that they have been tailored to their clinical parameters [28]. Beyond increasing patient compliance and efficacy of treatment plans, these devices are also able to generate digital records of rehabilitation sessions [39]. Access to digital records aids healthcare professionals, allowing them to dedicate more time to patient-centered activities. Furthermore, having detailed records always available has the potential to aid in the evaluation and possible modification of treatment plans.

Despite the many advantages that VR is projected to bring to the field of shoulder rehabilitation, the validity and effectiveness of kinematic intervention in virtual environments for orthopedic rehabilitation have only been explored by a few studies [3,27,28,29], none of which mention evaluating the performance of VR applications against pre-established criteria like clinical guidelines and/or staged ROM goals. Hence, the performed investigation compared the movements performed during different levels of the VR app against the gold-standard values using a motion capture system.

The analysis was approached with a methodology similar to that of a study published in 2015 in which authors combined motion tracking and virtual reality, using Oculus Rift DK2, to create a video game [32]. The game explains certain movements to patients that must then be replicated; patient movements are simultaneously tracked as they are being performed to evaluate whether they are being carried out properly [32]. However, the study did not subject patients to clinical experimentation; instead, they had experts in the field test the products and provide an evaluation. Thus, although there is a similarity in methodology, results cannot be compared.

Although present results are currently stand alone, given that, to our knowledge, the literature has not yet produced similar findings, the VR app seems to have a good performance when compared to gold-standard values measured via motion tracking. In terms of the first and second series of flexion, the total inferior MAE did not exceed 6.4, excluding one case in which the value was equivalent to 12.3. Regarding the number of times inferior flexion conditions were not met by the five volunteers, only once did the value exceed 8. For the superior flexion condition, volunteers performed with a margin of error only 6 times out of 30, while for the remaining 24 trials, volunteers had no individual MAE value. For inferior and superior conditions for abduction, volunteers performed no errors in levels 2 and 3, respectively. In levels 3 and 4, for inferior conditions, the number of times conditions were not met was slightly higher reaching a value of 22, while the total inferior MAE did not exceed 8.6. Overall results for flexion and abduction seem promising, with patients often reaching the gold-standard goal.

Experimentally internal and external rotation were found to have similar values for total MAE as those for flexion and abduction; however, the number of times patients did not meet conditions was higher compared to the other trials. This discrepancy is attributed to the fact that patients were unable to perform an isolated shoulder rotation. Instead, they aided the internal and external rotation of the shoulder by moving their trunk. Overall, more evidence is necessary to definitively confirm that VR rehabilitation programs can in fact have patients reach the same gold standard of movements as can be carried out with traditional rehabilitation programs. Nevertheless, current findings point to this being a possibility in the near future, and further exploration into the topic is necessary to better evaluate the effectiveness of these applications and their development into very effective therapeutic tools.

The growing impact of VR on shoulder rehabilitation is undeniable, as seen by the promising results of the present study, as well as the several new experimental trials working on the development of these VR applications. One double-blinded pilot study found that manipulated virtual real-time presentation of a subject’s movements during shoulder exercises promoted greater active flexion ROM [3]. Another study investigated the influence of game features and practice type, task-oriented or imitation-oriented, on shoulder muscle activity [40]. Findings showed that task-oriented practice elicited more intensive shoulder movements and muscular efforts [40]. These findings combined continue to support the hypothesis that VR is going to enhance patient adherence to rehabilitation programs, increase the ability of professionals to ‘personalize’ rehabilitation for each patient, and, most importantly, have the potential to improve patient functional outcomes.

A small cohort of healthy volunteers was included in this study, which could have a negative impact on the external validity and generalizability of data, in addition to not providing very strong statistical significance to present findings. Despite the negative effects of using a small cohort of healthy volunteers, given the novel topic and the little literature available, present results can serve as a starting point for further, larger-scale investigations, providing support for future research on the topic. Factors such as age, pathology, level of pain, and emotional state may affect the use of VR applications. Investigating the effect of these factors on the use of VR devices while performing rehabilitation protocols was beyond the scope of the present study. Nevertheless, in future studies, stratification for these factors can be very useful in analyzing how these factors could affect the use of VR devices among patients and further explore the validity of VR apps for shoulder rehabilitation after arthroscopic rotator cuff repair.

## 5. Conclusions

Our study assessed the performance of a custom shoulder rehabilitation VR app by comparing movements performed by volunteers at different levels of the program with a stereophotogrammetric system considered the gold standard. Data is promising, and volunteers were able to reach goal conditions more often than not. Mean error values were generally low, with some exceptions. It is important to note that internal and external rotation trials had higher numbers of unmet conditions at each level, which was attributed to unwanted compensatory movements of the trunk. Despite these promising results, more of the literature comparing VR applications utilized for shoulder rehabilitation with gold-standard clinical parameters is necessary. Nevertheless, the present study contributes to a body of literature that continues to encourage the application of VR to rehabilitation programs, given its ability to improve patient satisfaction and, in time, possibly to improve patient functional outcomes.

## Figures and Tables

**Table 1:** Movements, ROM, and repetitions for each level included in the immersive virtual reality application.

Level		Flexion 1	Flexion 2	Abduction	External Rotation	Internal Rotation
L1	Repetitions	10	10	10	8	-
	ROM	60°–90°	60°–90°	≤45°	≤20°	–
L2	Repetitions	10	10	10	8	8
	ROM	90°–120°	90°–120°	45°–80°	20°–30°	≤20°
L3	Repetitions	10	10	10	10	10
	ROM	130°–155°	130°–155°	80°–120°	30°–45°	20°–50°
L4	Repetitions	12	12	12	12	12
	ROM	≥140°	≥140°	≥120°	≥45°	≥50°

**Table 2:** Results for the first series of flexion for each level (MAE_inf_ e MAE_sup_ are the mean absolute errors corresponding to each volunteer; N_inf_ and N_sup_ correspond to the number of times the inferior and superior conditions were not met, respectively; MAE_TOT,inf_ and MAE_TOT,sup_ are the total mean absolute errors related to the inferior and superior conditions, respectively).

Level	Volunteer	MAE_inf_	N_inf_	MAE_TOT,inf_	MAE_sup_	N_sup_	MAE_TOT,sup_
L1	V1	1.0	8	6.4	✔	5	2.9
	V2	3.3			✔		
	V3	2.7			3.4		
	V4	✔			2.6		
	V5	10.2			✔		
L2	V1	2.3	15	5.1	✔	0	✔
	V2	5.5			✔		
	V3	2.6			✔		
	V4	2.8			✔		
	V5	7.9			✔		
L3	V1	✔	4	1.3	✔	3	8.6
	V2	✔			✔		
	V3	0.9			✔		
	V4	1.7			✔		
	V5	✔			8.6		
L4	V1	✔	1	0.8	-	-	-
	V2	✔			-		
	V3	0.8			-		
	V4	✔			-		
	V5	✔			-		

✔: condition satisfied.

**Table 3:** Results for the second series of flexion for each level (MAE_inf_ and MAE_sup_ are the mean absolute errors corresponding to each volunteer for the inferior and superior conditions, respectively; N_inf_ and N_sup_ correspond to the number of times the inferior and superior conditions were not met, respectively; MAE_TOT,inf_ and MAE_TOT,sup_ are the total mean absolute errors related to the inferior and superior conditions, respectively).

Level	Volunteer	MAE_inf_	N_inf_	MAE_TOT,inf_	MAE_sup_	N_sup_	MAE_TOT,sup_
L1	V1	6.8	6	4.6	✔	6	10.5
	V2	2.8			✔		
	V3	✔			✔		
	V4	✔			10.5		
	V5	2.3			✔		
L2	V1	8.0	8	12.3	✔	7	
	V2	15.4			✔		
	V3	✔			✔		9.2
	V4	✔			9.2		
	V5	13			✔		
L3	V1	✔	2	4.8	✔	2	2.1
	V2	7.9			✔		
	V3	✔			✔		
	V4	1.7			✔		
	V5	✔			2.1		
L4	V1	✔	8	2.6	-	-	-
	V2	2.6			-		
	V3	✔			-		
	V4	✔			-		
	V5	✔			-		

✔: condition satisfied.

**Table 4:** Results for abduction for each level (MAE_inf_ and MAE_sup_ are the mean absolute errors corresponding to each volunteer for the inferior and superior conditions, respectively; N_inf_ and N_sup_ correspond to the number of times the inferior and superior conditions were not met, respectively; MAE_TOT,inf_ and MAE_TOT,sup_ are the total mean absolute errors related to the inferior and superior conditions, respectively).

Level	Volunteer	MAE_inf_	N_inf_	MAE_TOT,inf_	MAE_sup_	N_sup_	MAE_TOT,sup_
L1	V1	-	-	-	5.9	14	6.1
	V2	-			7.8		
	V3	-			✔		
	V4	-			✔		
	V5	-			1.2		
L2	V1	✔	0	✔	✔	4	
	V2	✔			4.7		
	V3	✔			✔		4.7
	V4	✔			✔		
	V5	✔			✔		
L3	V1	4.0	15	8.6	✔	0	✔
	V2	✔			✔		
	V3	11.4			✔		
	V4	8.2			✔		
	V5	8.0			✔		
L4	V1	6.6	22	6.6	-	-	-
	V2	5.8			-		
	V3	5.9			-		
	V4	10.8			-		
	V5	7.0			-		

✔: condition satisfied.

**Table 5:** Results for external rotation for each level (MAE_inf_ and MAE_sup_ are the mean absolute errors corresponding to each volunteer for the inferior and superior conditions, respectively; N_inf_ and N_sup_ correspond to the number of times the inferior and superior conditions were not met, respectively; MAE_TOT,inf_ and MAE_TOT,sup_ are the total mean absolute errors related to the inferior and superior conditions, respectively).

Level	Volunteer	MAE_inf_	N_inf_	MAE_TOT,inf_	MAE_sup_	N_sup_	MAE_TOT,sup_
L1	V1	-	-	-	11.5	32	6.5
	V2	-			3.4		
	V3	-			1.9		
	V4	-			5.7		
	V5	-			7.9		
L2	V1	✔	1	2.3	2.9	12	
	V2	✔			1.7		
	V3	✔			✔		2.6
	V4	2.3			✔		
	V5	✔			2.6		
L3	V1	✔	16	6.3	2.6	5	2.6
	V2	✔			✔		
	V3	1.3			✔		
	V4	10.3			✔		
	V5	0.1			✔		
L4	V1	1.1	26	19.6	-	-	-
	V2	✔			-		
	V3	15.8			-		
	V4	26.6			-		
	V5	✔			-		

✔: condition satisfied.

**Table 6:** Results for internal rotation for each level (MAE_inf_ and MAE_sup_ are the mean absolute errors corresponding to each volunteer for the inferior and superior conditions, respectively; N_inf_ and N_sup_ correspond to the number of times the inferior and superior conditions were not met, respectively; MAE_TOT,inf_ and MAE_TOT,sup_ are the total mean absolute errors related to the inferior and superior conditions, respectively).

Level	Volunteer	MAE_inf_	N_inf_	MAE_TOT,inf_	MAE_sup_	N_sup_	MAE_TOT,sup_
L2	V1	-	-	-	17.3	33	9.8
	V2	-			2.8		
	V3	-			8.0		
	V4	-			0.2		
	V5	-			12.4		
L3	V1	✔	6	2.0	✔	0	✔
	V2	1.9			✔		
	V3	✔			✔		
	V4	2.2			✔		
	V5	✔			✔		
L4	V1	✔	30	8.0	-	-	-
	V2	4.3			-		
	V3	7.6			-		
	V4	10.7			-		
	V5	3.1			-		

✔: condition satisfied.

## Data Availability

Data available from the corresponding author on reasonable request.
